# Recovery of Anthocyanins from Passion Fruit Epicarp for Food Colorants: Extraction Process Optimization and Evaluation of Bioactive Properties

**DOI:** 10.3390/molecules25143203

**Published:** 2020-07-14

**Authors:** Bejaoui Ghada, Eliana Pereira, José Pinela, Miguel A. Prieto, Carla Pereira, Ricardo C. Calhelha, Dejan Stojković, Marina Sokóvić, Khalil Zaghdoudi, Lillian Barros, Isabel C. F. R. Ferreira

**Affiliations:** 1 Centro de Investigação de Montanha (CIMO), Instituto Politécnico de Bragança, Campus de Santa Apolónia, 5300-253 Bragança, Portugal; bejghada94@gmail.com (B.G.); jpinela@ipb.pt (J.P.); carlap@ipb.pt (C.P.); calhelha@ipb.pt (R.C.C.); iferreira@ipb.pt (I.C.F.R.F.); 2 Department of Chemical Engineering, Tunisia Private University (ULT), 32 Bis Av. Kheireddine Pacha, Tunis 1002, Tunisia; khalilo.zg@gmail.com; 3 Nutrition and Bromatology Group, Faculty of Food Science and Technology, University of Vigo, Ourense Campus, E32004 Ourense, Spain; michaelumangelum@gmail.com; 4 Institute for Biological Research “Siniša Stanković”—National Institute of Republic of Serbia, University of Belgrade, Bulevar Despota Stefana 142, 11000 Belgrade, Serbia; dejanbio@ibiss.bg.ac.rs (D.S.); mris@ibiss.bg.ac.rs (M.S.)

**Keywords:** *Passiflora edulis* Sims, anthocyanins, extraction optimization, natural colorants, antioxidant activity, oxidative hemolysis, antimicrobial activity

## Abstract

The potential of passion fruit (*Passiflora edulis* Sims) epicarp to produce anthocyanin-based colorants with bioactive properties was evaluated. First, a five-level three-factor factorial design coupled with response surface methodology was implemented to optimize the extraction of anthocyanins from dark purple epicarps. The extraction yield and cyanidin-3-*O*-glucoside content were used as response criteria. The constructed models were fitted to the experimental data and used to calculate the optimal processing conditions (*t* = 38 min, *T* = 20 °C, *S* = 0% ethanol/water (*v/v*) acidified with citric acid to pH 3, and *R_S/L_* = 50 g/L) that lead to maximum responses (3.4 mg/g dried epicarp and 9 mg/g extract). Then, the antioxidant, antimicrobial, and cytotoxic activities of anthocyanin extracts obtained using the optimized method and a conventional extraction method were evaluated in vitro. The extract obtained by the optimized method revealed a higher bioactivity, in agreement with the higher cyanidin-3-*O*-glucoside content. This study highlighted the coloring and bioactive potential of a bio-based ingredient recycled from a bio-waste, which promotes a sustainable bioeconomy in the agri-food sector.

## 1. Introduction

The food industry is a highly regulated sector in Europe, notably in terms of safety and hygiene, with the priority of reducing the environmental impact, especially the production of bio-waste [1]. In this regard, the European Waste Framework Directive has required Member States to take measures to encourage the selective collection of bio-waste for composting, digestion, and treatment, and the use of environmentally safe materials produced from this bio-waste [2].

In the fruit processing industry, the amount of bio-waste obtained during transformation is generally between 30–50%. Seeds, pulp, bagasse, and peel are the most common bio-waste resulting from these operations, which are often discarded or used as fertilizers and in animal feed [3]. However, substantial evidence has shown that all of this waste is rich in bioactive phytochemicals, which has attracted a great deal of interest among researchers and industry in promoting their recovery and recycling within the food chain through the creation of high value-added bio-based products [4,5].

One sector that generates a considerable amount of bio-waste and by-products is the manufacture of juices from exotic fruits such as passion fruit (*Passiflora edulis* Sims), which are among the most important fruits for the juice industry in the world market and have a high percentage of inedible or unusable parts [4]. The purple passion fruit is a delicious fruit with nutritional and medicinal properties [6]. During processing, the epicarp is generally discarded as a solid bio-waste, which constitutes approximately half of the fruit weight [7]. This purple part of the fruit peel is rich in bioactive constituents such as flavonoids, phenolic acids, and pigments like anthocyanins [8].

Anthocyanins have a high potential to be used as natural colorants in the food industry due to their attractive orange, red, blue, and purple colors, and water solubility, which allows their incorporation into aqueous food systems [9]. However, anthocyanins stand out not only for their coloring potential, but also for their health-promoting effects [10,11]. According to some authors, the health benefits are linked with antioxidant, antimicrobial, and anti-ulcer properties, and the ability to maintain normal vascular permeability [10,11]. In addition, anthocyanins isolated and purified from fruit and vegetables may be useful in the treatment of chronic diseases, particularly, type 2 diabetes. However, the antioxidant activity has been the most significant bioactivity attributed to anthocyanin-rich extracts [12]. 

Aiming to promote the reuse of passion fruit epicarp in the industrial sector as a raw material for obtaining natural colorants, this study was performed to optimize the extraction of anthocyanins using the response surface methodology (RSM). The anthocyanin levels used as a response variable in the optimization process were obtained by liquid chromatography coupled with mass spectrometry (HPLC-DAD-ESI/MS). The bioactive potential of anthocyanin extracts obtained under the optimized processing conditions and by a conventional extraction method were also evaluated in vitro and compared for their antioxidant, antimicrobial and cytotoxic properties.

## 2. Results and Discussion

### 2.1. Colour Parameters of Fresh and Dried Epicarp

The color parameters of the plant matrices may reflect the type of molecules present in their composition. Thus, in this study, the color of the passion fruit peel was measured in order to preliminarily assess the typology of the coloring compounds, as well as the color tone and intensity. It was also important to understand the interference of the dehydration process in relation to the peel color, that is, if there was a significant degradation of the coloring pigments. The results of the color analysis of the purple passion fruit epicarp are presented in Table 1. The parameter L* denotes lightness, a* corresponds to chromaticity on a green (−) to red (+) axis, and b* to chromaticity on a blue (−) to yellow (+) axis. As observed, the freeze drying process affected the epicarp color. The L* parameter did not suffer a significant change, with values close to 34 in the CIE L*a*b* color space, while the other two parameters were significantly (*p* < 0.05) affected by drying. A higher a* value was recorded on the dried epicarp, thus translating a more intense red color. In turn, the same sample showed a lower b* value. These changes in color coordinates indicate that the passion fruit epicarp acquired a shade closer to purple after lyophilization. To better visualize these color differences, data were converted into RGB values and the obtained colors are shown in Table 1.

### 2.2. Anthocyanin Profile of the Epicarp Extract

Data of the anthocyanin analysis in the passion fruit epicarp hydroethanolic extract obtained by the conventional solid-liquid extraction are shown in Supplementary Materials, Table S1. The compound identification was made based on the retention time, UV-Vis spectrum, and MS fragmentation patterns. The analysis revealed the presence of one anthocyanin (Figure S1), identified as cyanidin-3-*O*-glucoside by chromatographic data comparison with those of the commercial standard. It has a [M+H]^+^ ion at *m/z* 449 and a MS^2^ fragment at *m/z* 287 (Table S1). In this study, the conventional extraction method, previously described by Jabeur et al. [12], where solid-liquid extraction is performed twice (1 h + 1 h) with 80% ethanol acidified with 0.05% citric acid as the solvent, yielded 34% extract that contained 8.3 ± 0.1 mg of cyanidin-3-*O*-glucoside/g extract. Therefore, it was possible to recover 2.82 mg of cyanidin-3-*O*-glucoside per gram of dried epicarp.

Kidøy et al. [13] also reported cyanidin-3-*O*-glucoside (97%) and small amounts of cyanidin-3-*O*-(6-malonylglucoside) (2%) and pelargonidin-3-*O*-glucoside (1%) in a passion fruit peel extract obtained using methanol acidified with 2% trifluoroacetic acid. In another study, Jiménez et al. [14] identified cyanidin-3-*O*-*β*-*D*-glucopyranoside in a peel extract obtained with methanol acidified with acetic acid (19:1, *v/v*). The anthocyanins delphinidin-3,5-*O*-glucoside, cyanidin-3-*O*-glucoside and pelargonidin-3-*O*-glucoside, and the aglycones delphinidin and cyanidin were reported by Dos Reis et al. [15] in purple passion fruit peel homogenized with acidified methanol (1% HCl).

### 2.3. Optimization of the Extraction Process for Obtaining Anthocyanin-Rich Extracts

Although there are some studies on extraction of anthocyanins from passion fruit peel, there are few that detail the processing conditions that maximize their recovery. Moreover, the different qualitative and quantitative anthocyanin profiles of the different natural sources does not allow the direct extrapolation of the extraction conditions delineated for other matrices [16]. Therefore, the approach adopted to reduce costs (energy and solvent consumption) and optimize the extraction of cyanidin-3-*O*-glucoside from purple passion fruit epicarp was to use an RSM-coupled central composite design (CCD).

The CCD design presented in Table 2 was used to optimize the extraction conditions for anthocyanins considering the following independent variables and ranges: time, *t* (5–85 min); temperature, *T* (20–90 °C); and solvent proportion (% ethanol in water), *S* (0–100%). A comprehensive summary of the different steps carried out in the optimization study is illustrated in Supplementary Materials, Figure S2.

#### 2.3.1. Response Criteria for RSM Analysis

The response criteria used to optimize the anthocyanin extraction are shown in Table 3, namely the anthocyanin (cyanidin-3-*O*-glucoside) content in both dried epicarp (*Y*_1_) and extract (*Y*_2_) and the extraction yield (*Y*_1_/*Y*_2_). These response criteria are of interest to industrial sectors dealing with the recovering of high value-added molecules from plant materials or the production of bio-based ingredients, and provide information on the amount of plant material required to obtain a certain quantity of the desired compounds and the concentration of these natural compounds in the produced extracts.

According to the CCD design matrix in Table 3, it was possible to recover from 0.74 to 2.58 mg of cyanidin-3-*O*-glucoside from 1 g of dried epicarp (with runs 5 and 1, respectively), and to obtain from 2.84 to 9.34 mg of the same compound per gram of extract (with runs 5 and 2, respectively). The extraction yield was also affected and ranged from 12.1 to 44.6% (with runs 10 and 13, respectively).

#### 2.3.2. Model and Response Surface Analysis

The response values shown in Table 3 were fitted to a second-order polynomial equation to obtain the parametric values shown in Table 4, where the results of the statistical verification of the obtained models are also presented. The fitting procedure was made using nonlinear least-squares estimations, in which the non-significant terms were excluded; the significant ones were assessed at a 95% confidence level (α = 0.05). The parametric values reveal the complexity associated with the extraction process and reflect the expected variation in the response per unit change in factor value when all remaining variables are kept constant. The intercept is the overall average response of the RSM design matrix runs (Table 3).

The developed models had a non-significant lack-of-fit and were statistically validated and used to navigate the design space in the optimization steps.

**Figure 1:**
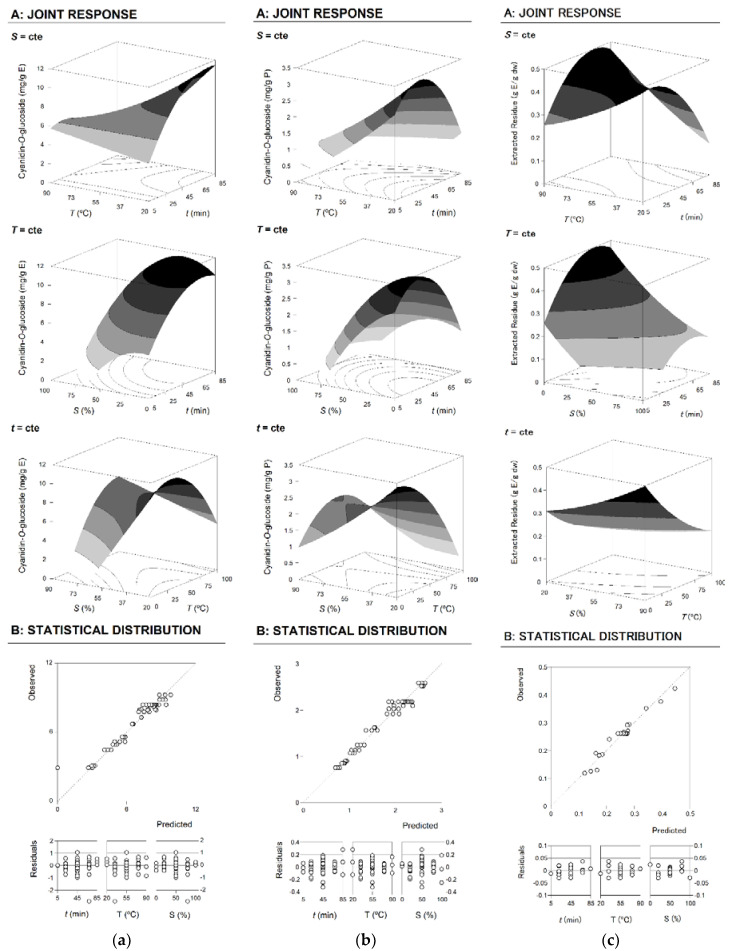
Graphical results for the response criteria (**a**) (mg anthocyanin/g dried epicarp), (**b**) (mg anthocyanin/g extract), and (**c**) (g extract/g dried epicarp). The 3D response surface graphs are shown in Part A. In each graph, the excluded variable is fixed at the individual optimum presented in Table 4. The goodness of fit is illustrated in Part B through the ability to simulate response changes between predicted and observed data and the residual distribution as a function of each variable.

**Figure 2:**
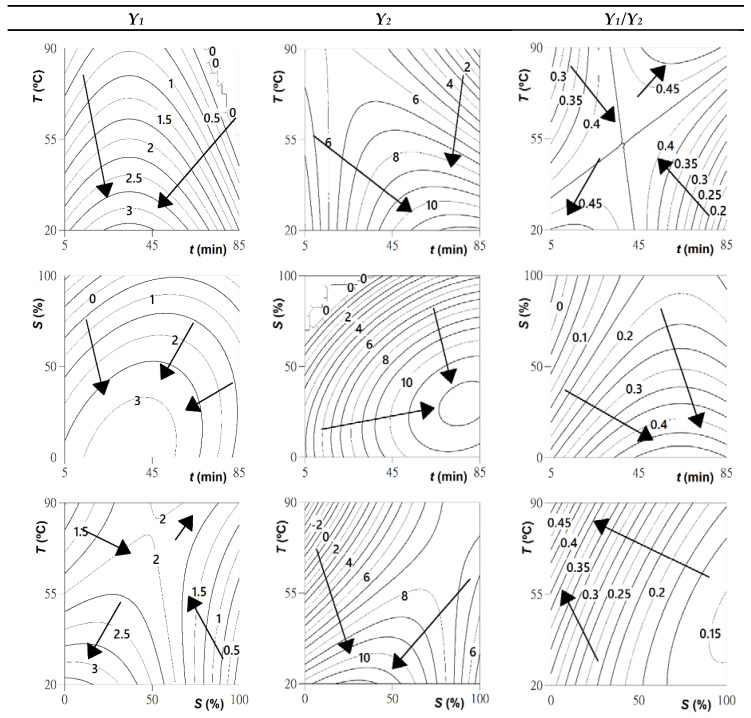
Projections in XY plane of the 3D response surfaces for the response criteria *Y*_1_ (mg anthocyanin/g dried epicarp), *Y*_2_ (mg anthocyanin/g extract), and *Y*_1_***/****Y*_2_ (g extract/g dried epicarp) that describe the most favorable extraction conditions. In each projection, the excluded variable is fixed at the individual optimum presented in Table 4.

Although parametric values show responses patterns, the best way to visually describe the effects of any independent variable in the recovery process of bioactive molecules is to generate 3D response surface plots, representing the combined effects of two variables and keeping the other variable constant. Figure 1 shows the 3D surface graphs for the three studied response criteria, and the corresponding 2D contour graphs focusing the optimal extraction points are represented in Figure 2. Both surface and contour graphs are in accordance with the parametric values derived from the multiple regression analysis presented in Table 4.

The 3D surface graphs translate the parametric results of Table 4 and visually represent the influence of the three independent variables involved in the process (Figure 1). Part A shows 3D response surfaces. In each graph, the combined effects of two variables were represented with the excluded variable positioned at its individual optimal value, which is shown in Table 4. In turn, part B illustrates the capability of the models to predict the obtained results and the residual distribution as a function of each variable. A good agreement between experimental and model-predicted values was observed, with R^2^ values higher than 0.95 in all cases (Table 4), which translates the percentage of variability explained by the models. In addition, the errors had constant variance, with the residuals scattered randomly around zero.

#### 2.3.3. Optimal Extraction Conditions and Verification of the Predictive Models

Once the models were statistically validated, they were used determine the optimal extraction conditions that maximize the individual and global responses. Table 4, part C, presents the optimal processing conditions that maximize the recovery of anthocyanins from passion fruit pericarp. It was possible to recover 3.4 ± 0.6 mg of cyanidin-3-*O*-glucoside from 1 g of plant material when processing it at 20 °C for 35 min and using 0% ethanol, while 78 ± 8 mg of the same anthocyanin were obtained per each gram of extract when processing the sample at 20 °C for 78 min and using 29% ethanol (*v/v*). In order to maximize the amount of anthocyanin in the extract and the recovery rate of the target compound from the dried epicarp, global processing conditions were also calculated (Table 4). These conditions (*t* = 38 min, *T* = 20 °C; *S* = 0% ethanol, *v/v*) allowed 9 ± 1 mg of cyanodin-3-*O*-glucoside/g of extract to be obtained and 3.4 ± 0.5 mg of the same anthocyanin/g of dried epicarp to be recovered, representing a higher extraction yield (37 ± 5%) than the conventional method. Although the improvements obtained with the optimized process are not extremely pronounced in terms of yield and anthocyanin contents compared to the conventional method, the optimized protocol stands out for requiring a much shorter extraction time (37 min vs. 2 h) and just water (vs. 80% ethanol) as extraction solvent. At the industrial level, these processing conditions are thus time-saving, economically attractive, and “greener” and therefore more desired and advantageous. These global conditions were then applied to obtain a new extract and thus evaluate the predictive model’s ability to predict the experimental results. The model was experimentally validated with a good correlation between model-predicted and experimental results.

### 2.4. Effect of the Solid-to-Liquid Ratio

The individual *R_S/L_* analysis was performed under the optimal conditions predicted by the models developed for each response variable (Table 4), and its effects were assessed at ratios between 5 and 100 g/L. The obtained *R_S/L_* responses were consistent with the results obtained in the RSM analysis, and were described by a linear relationship (data not shown). All experimental points were distributed around the equation with only one independent variable and, consequently, the dose-response effect was explained by the slope (*m*) and intercept (*b*) of the linear equation.

Although the lower *R_S/L_* yielded similar results, the responses decreased as the *R_S/L_* increased. The negative values of *m* show that increasing this ratio causes a decrease in extraction efficiency, resulting in a maximum extraction value at 5 g/L and a minimum at 100 g/L. However, the observed decrease was marked, meaning that the increase of 1 g/L implies the loss of a significant amount of anthocyanin per gram of extract. Such values produce losses of ~45% in the maximum experimental value tested, compared to the value extracted at 5 g/L. However, the economic advantages of working at 100 g/L may outweigh the possible benefits of extracting at the optimal *R_S/L_*. From the optimization point of view, it was decided that the value used would be around 50 g/L.

### 2.5. Bioactive Properties of Anthocyanin Extracts Obtained by the Conventional and Optimized Methods

#### 2.5.1. Antioxidant Activity

The antioxidant activity of the anthocyanin extracts obtained from passion fruit epicarp using the optimized (optimal extract) and conventional (normal extract) methods was evaluated through its capacity to prevent the formation of thiobarbituric acid reactive substance (TBARS) and the oxidative hemolysis. The results of both in vitro assays are present in Table 5. As observed, the antioxidant activity of the extracts differed significantly (*p* < 0.05), and the highest EC_50_ and IC_50_ values were achieved with the normal extract. Thus, the extract obtained under optimal conditions stood out with greater ability to inhibit the TBARS formation and oxidative hemolysis, being suitable for lipid peroxidation inhibition in food products. This result can be explained by the higher concentration of cyanidin-3-*O*-glucoside found in the optimal extract; nevertheless, other non-identified compounds may also contribute to this bioactivity.

The antioxidant properties of passion fruit extracts have been reported by other authors, which measured this activity by different in vitro assays, such as ABTS, DPPH and FRAP [17,18,19], and pointed out a correlation between total phenolics and antioxidant capacity.

#### 2.5.2. Antimicrobial Activity

The results of the antimicrobial activity of the two extracts are presented in Table 5. The optimal extract showed a slightly higher antimicrobial activity than the normal extract against *Listeria monocytogenes* and *Escherichia coli*, with a MIC of 4 mg/mL versus 8 mg/mL, respectively. Regarding the MBC values, 8 mg/mL of optimal extract was enough to kill the tested bacteria, while a concentration above 8 mg/mL of normal extract was generally required. The optimal extract was therefore the most effective one.

Among the six fungi tested, *Penicillium ochrochloron* was the most sensitive to the optimal extract, with 1 mg/mL being required to inhibit and kill this fungus (Table 5), followed by *Trichoderma viride* and *Aspergillus niger* (MICs = 4 mg/mL). The other strains had MIC values of 8 mg/mL for this extract, but still lower than those required of normal anthocyanin extract (>8 mg/mL).

#### 2.5.3. Cytotoxic Activity

The cytotoxic activity of the tested passion fruit epicarp extracts against four tumor cell lines (NCI-H460, MCF-7, HepG2 and HeLa) and primary PLP2 cells are presented in Table 5. The normal extract showed no cytotoxic activity against the tumor cell lines at the tested concentrations (GI_50_ > 400 μg/mL), while the anthocyanin extracted obtained under optimized processing conditions exhibited weak cytotoxicity to HepG2 cells, with a GI_50_ value of 363 ± 15 µg/mL. Interestingly, none of the extracts showed hepatotoxicity up to a concentration of 400 μg/mL to non-tumor PLP2 cells. To the best of the authors’ knowledge, there are no studies reporting the cytotoxicity or hepatotoxicity of passion fruit extracts.

## 3. Materials and Methods

### 3.1. Sample Preparation

Fresh passion fruit samples were supplied by the company KiwiCoop from Oliveira do Bairro, Portugal. The epicarp was separated from the pulp and then freeze-dried (FreeZone 4.5, Labconco, Kansas City, MO, USA) and reduced to powder (~20 mesh), which was homogenized to obtain a representative sample that was kept at −80 °C.

### 3.2. Determination of Colour in Fresh and Dried Epicarp

Color was measured on fresh and dried epicarps with a CR-400, Konica Minolta Sensing colorimeter (Osaka, Japan). Measurements were performed in the CIELAB color space as described by López et al. [20]. Data were processed with Spectra Magic Nx software (CM-S100W 2.03.0006, Konica Minolta).

### 3.3. Extraction and Analysis af Anthocyanins from Dried Epicarp

A conventional extraction was performed as formerly described by Jabeur et al. [12]. Powdered epicarp (1 g) underwent solid-liquid extraction twice (1 h + 1 h) with 80% ethanol acidified with 0.05% citric acid (20 mL). After filtering the supernatant, ethanol was separated in a Büchi R-210 rotary evaporator and water was freeze-dried.

For analysis of anthocyanins, the extract was dissolved in 20% ethanol at 5 mg/mL and filtered through a 0.2-μm syringe filter disk for injection in the HPLC-DAD-ESI/MS system formerly described by Bessada et al. [21], which also summarized the chromatographic conditions and the identification and quantification procedures. The calibration curve (y = 243287x − 1 × 10^6^; *r*^2^ = 0.9953) used in the quantification was constructed with the standard cyanidin-3-*O*-glucoside (Extrasynthèse, Genay, France). The results were given in mg/g extract.

### 3.4. Experimental Design

A CCD of 20 runs was applied to optimize the anthocyanin extraction from passion fruit epicarp using RSM. The natural and coded values of the independent variables *X*_1_ (time (*t*), min), *X*_2_ (temperature (*T*), °C) and *X*_3_ (solvent (*S*) or ethanol proportion, % (*v*/*v*)) are presented in Table 2.

### 3.5. Extraction Method

Extractions were made as described by Pinela et al. [16]. Powdered epicarp samples (1 g) were mixed with 20 mL of solvent (ethanol/water mixtures acidified with 0.05% citric acid, until pH 3) and processed by stirring at 500 rpm according to the CCD design shown in Table 2, which combines five levels of *t* (5–85 min), *T* (20–90 °C), and *S* (0–100%). After processing, the mixtures were centrifuged (Centurion K24OR, West Sussex, UK) at 500 rpm for 20 min and the supernatants were filtered through filter paper to determine the extraction yield and anthocyanin content.

### 3.6. Extraction Process Optimization by Response Surface Methodology

The extraction yield and anthocyanin content in the filtrates were analyzed and used as response criteria. Anthocyanins were quantified as described in Section 2.3. and the extract weight (%, *w*/*w*) was determined gravimetrically by evaporating the solvent from the supernatants [16]. Three response criteria were used for RSM optimization, namely: *Y*_1_, anthocyanin content in the dried epicarp (mg/g dried epicarp); *Y*_2_, anthocyanin content in the extract (mg/g extract); and *Y*_1_/*Y*_2_, extraction yield (g extract/g dried epicarp).

The response surface models were fitted to the experimental data using the second-order polynomial equation described by Pinela et al. [22]. All fitting procedures, coefficient estimates and statistical analysis were performed as explained by Prieto and Vázquez [23].

### 3.7. Analysis of the Solid-to-Liquid Ratio

The solid-to-liquid ratio (*R_S/L_*) was studied after optimizing the extraction conditions for the other three variables. The *R_S/L_* effects were depicted using a linear equation in which a positive slope value indicates an increase in extraction efficiency (Pinela et al., 2019).

### 3.8. Production of Anthocyanin Extract under Optimal Extraction Conditions and Verification of the Predictive Models

An anthocyanin-rich extract was produced from passion fruit epicarp applying the optimized processing conditions previously established. The sample (1 g) was mixed with 100% water (20 mL) acidified with 0.05% citric acid (until pH 3) and processed at 20 °C for 37.5 min. After centrifugation, the supernatant was filtered and the solvent eliminated to obtain a dry extract. Thereafter, the anthocyanin content was determined to evaluate the model predictive capacity.

### 3.9. Evaluation of bioactive Properties of the Anthocyanin Extracts Obtained by the Conventional and Optimized Methods

#### 3.9.1. Antioxidant Activity

The TBARS formation inhibition and oxidative hemolysis inhibition (OxHLIA) assays were performed as described by Lockowandt et al. [24]. The extract’s capacity to inhibit the formation of TBARS was assessed using porcine brain cells as oxidizable substrates and the results were given as EC_50_ values (µg/mL). In turn, the extracts capacity to inhibit the oxidative hemolysis was tested using sheep erythrocytes and the results were given as IC_50_ values (μg/mL) for a Δ*t* of 60 min. Trolox was the positive control.

#### 3.9.2. Antimicrobial Activity

The antibacterial activity was tested against *Escherichia coli*, *Salmonella typhimurium*, *Enterobacter cloacae*, *Staphylococcus aureus*, and *Listeria monocytogenes* as described by Soković et al. [25], using ampicillin as positive control. The antifungal activity against *Aspergillus fumigatus*, *Aspergillus versicolor*, *Aspergillus niger*, *Penicillium funiculosum*, *Penicillium ochrochloron*, and *Trichoderma viride* was evaluated as described by Soković and van Griensven [26], using ketoconazol as positive control. The results were given as minimum inhibitory, bactericidal, and/or fungicidal concentrations (MIC, MBC, and MFC, respectively, mg/mL).

#### 3.9.3. Cytotoxic Activity

The cytotoxicity against HeLa (cervical carcinoma), HepG2 (hepatocellular carcinoma), MCF-7 (breast adenocarcinoma), and NCI-H460 (non-small-cell lung cancer) cells was tested by the sulforhodamine B assay [27]. Non-tumor PLP2 cells were used to evaluate the hepatotoxic activity. Ellipticine was the positive control. The results were given as GI_50_ values.

### 3.10. Statistical Analysis

Color and bioactivity analyses were made in triplicate. SPSS Statistics (23.0, IBM Corp., Armonk, NY, USA) was used for data analysis. A Student’s *t*-test was performed to evaluate statistical differences between the two anthocyanin extracts (*p* < 0.05). 

## 4. Conclusions

The coloring and bioactive potential of a bio-based ingredient produced from the dark purple epicarp of passion fruit was evaluated. The dehydrated epicarp used for recovery of anthocyanins had a more intense red color (higher a* value) than the fresh material, which demonstrated the suitability of freeze-drying. For extraction optimization, *t*, *T*, and *S* were combined in a CCD design coupled with RSM, using the extraction yield and cyanidin-3-*O*-glucoside levels as dependent variables. The developed models were successfully fitted to the experimental data and used to determine the optimal processing conditions that lead to maximum responses, namely anthocyanin content recovered from the epicarp and obtained in the extract. The *R_S/L_* analysis showed that 50 g/L is a suitable ratio. The extract obtained by the optimized method revealed a higher bioactivity (TBARS inhibition, antihemolytic, antimicrobial, and antifungal activities and cytotoxicity against cervical carcinoma), in agreement with the higher cyanidin-3-*O*-glucoside content. Therefore, it is concluded that passion fruit epicarp is a sustainable source of cyanidin-3-*O*-glucoside and can be used to produce bioactive colorants.

## Figures and Tables

**Table 1:** Color parameters of fresh and dried passion fruit epicarp.

Samples	L* (Lightness)	a* (Redness)	b* (Yellowness)	RGB Colour
Fresh epicarp	34.2 ± 0.9	6.9 ± 0.3	5.1 ± 0.2	
Dried epicarp	34.3 ± 1.5	16.0 ± 0.6	2.8 ± 0.1	
***p*>-value**	0.761	<0.01	<0.01	

RGB values: fresh epicarp: red—89, green—67, blue—74; dried epicarp: red—97, green—62, blue—77.

**Table 2:** Natural and coded values of the independent variables used in the factorial design.

Coded Values	Natural Values		
	*t* (min)	*T* (°C)	*S* (%)
−1.68	5	20	0
−1	21.2	34.2	20.3
0	45	55	50
+1	21.2	75.8	79.7
+1.68	85	90	100

**Table 3:** Experimental results obtained under the conditions of the CCD design matrix for the responses *Y*_1_ (mg anthocyanin/g dried epicarp), *Y*_2_ (mg anthocyanin/g extract) and *Y*_3_ (*Y*_1_/*Y*_2_, g extract/g dried epicarp) used in the anthocyanin extraction optimization process by RSM.

Run	Coded Values			Natural Values			Experimental Responses		
	*X*_1_	*X*_2_	*X*_3_	*X*_1_: *t*	*X*_2_: *T*	*X*_3_: *S*	*Y*_1_	*Y*_2_	*Y*_1_/*Y*_2_
				min	°C	%	mg Anthocyanin/g Dried Epicarp	mg Anthocyanin/g Extract	g Extract/g Dried Epicarp
1	−1	−1	−1	21.2	34.2	20.3	2.58	6.54	0.395
2	−1	−1	+1	21.2	34.2	79.7	1.96	9.34	0.210
3	−1	+1	−1	21.2	75.8	20.3	1.48	5.41	0.274
4	−1	+1	+1	21.2	75.8	79.7	1.06	3.11	0.342
5	+1	−1	−1	68.8	34.2	20.3	0.74	2.84	0.262
6	+1	−1	+1	68.8	34.2	79.7	1.25	7.51	0.166
7	+1	+1	−1	68.8	75.8	20.3	1.13	7.88	0.143
8	+1	+1	+1	68.8	75.8	79.7	1.55	8.39	0.185
9	−1.68	0	0	5	55	50	0.87	4.98	0.174
10	+1.68	0	0	85	55	50	0.88	7.28	0.121
11	0	−1.68	0	45	20	50	2.57	9.12	0.282
12	0	+1.68	0	45	90	50	2.17	7.84	0.277
13	0	0	−1.68	45	55	0	1.95	4.36	0.446
14	0	0	+1.68	45	55	100	0.93	5.74	0.162
15	0	0	0	45	55	50	2.14	8.56	0.251
16	0	0	0	45	55	50	2.27	8.38	0.271
17	0	0	0	45	55	50	2.06	7.93	0.260
18	0	0	0	45	55	50	2.27	8.27	0.274
19	0	0	0	45	55	50	2.27	8.46	0.268
20	0	0	0	45	55	50	2.06	8.55	0.241

**Table 4:** Parametric results obtained for the responses *Y*_1_ (mg anthocyanin/g dried epicarp), *Y*_2_ (mg anthocyanin/g extract), and *Y*_3_ (*Y*_1_/*Y*_2_, g extract/g dried epicarp) used to optimize the extraction of anthocyanins from passion fruit epicarp by RSM (part A). Part B shows the statistical information of the fitting procedure. Part C presents the variable conditions in natural values that lead to optimal response values.

		*Y*_1_		*Y*_2_		*Y*_1_/*Y*_2_	
**(A) Parametric information**							
Intercept	*b*_0_	2.18	±0.06	8.4	±0.2	0.26	±0.01
Linear effect	*b*_1_	ns		0.7	±0.1	−0.02	±0.01
	*b*_2_	−0.15	±0.04	−0.3	±0.1	ns	
	*b*_3_	−0.30	±0.04	0.3	±0.1	−0.07	±0.01
Quadratic effect	*b*_11_	−0.47	±0.04	−0.8	±0.1	−0.04	±0.01
	*b*_22_	0.06	±0.04	ns		ns	
	*b*_33_	−0.27	±0.04	−1.2	±0.1	0.02	±0.01
Interactive effect	*b*_12_	ns		−1.2	±0.2	0.05	±0.01
	*b*_13_	0.25	±0.05	0.6	±0.2	ns	
	*b*_23_	0.34	±0.05	1.7	±0.2	ns	
**(B) Statistical information**							
R^2^		0.9677		0.9546		0.9542	
R^2^ adjusted		0.962		0.951		0.951	
**(C) Optimal variable conditions for response maximization**							
Individual conditions	Time (min)	35	±2	78	±8	64	±8
	Temperature (°C)	20	±4	20	±4	90	±3
	Solvent (%)	0	±2	29	±4	0.0	±0.9
	Response:	3.4	±0.6	12	±2	0.29	±0.04
Global conditions	Time (min)	38 ± 4					
	Temperature (°C)	20 ± 2					
	Solvent (%)	0 ± 2					
	Response:	3.4	±0.5	9	±1	0.37	±0.05

**Table 5:** Antioxidant, antimicrobial and cytotoxic activities of the anthocyanin extracts obtained from passion fruit epicarp using the optimized and conventional extraction methods.

	Optimal Extract		Normal Extract		Positive Control	
**Antioxidant activity**					Trolox	
TBARS (EC_50_, µg/mL)	115 ± 3		136 ± 4		20.4 ± 0.5	
OXHLIA (IC_50_, µg/mL)	78 ± 3		144 ± 4		5.4 ± 0.3	
Cytotoxicity to tumor cells					Ellipticine	
NCI-H460 (GI_50_, µg/mL)	>400		>400		1.03 ± 0.09	
MCF-7 (GI_50_, µg/mL)	>400		>400		1.1 ± 0.2	
HepG2 (GI_50_, µg/mL)	363 ± 15		>400		1.1 ± 0.2	
HeLa (GI_50_, µg/mL)	>400		>400		1.91 ± 0.06	
**Hepatotoxicity**					Ellipticine	
PLP2 (GI_50_, µg/mL)	>400		>400		3.2 ± 0.7	
**Antibacterial activity**					Ampicillin	
	MIC (mg/mL)	MBC (mg/mL)	MIC (mg/mL)	MBC (mg/mL)	MIC (mg/mL)	MBC (mg/mL)
*Staphylococcus aureus*	8.00	8.00	8.00	>8.00	0.012	0.025
*Listeria monocytogenes*	4.00	8.00	8.00	>8.00	0.40	0.50
*Escherichia coli*	4.00	8.00	8.00	8.00	0.40	0.50
*Enterobacter cloacae*	8.00	8.00	8.00	>8.00	0.006	0.012
*Salmonella typhimurium*	8.00	8.00	8.00	>8.00	0.75	1.20
**Antifungal activity**					Ketoconazol	
	MIC (mg/mL)	MFC (mg/mL)	MIC (mg/mL)	MFC (mg/mL)	MIC (mg/mL)	MFC (mg/mL)
*Aspergillus fumigatus*	8.00	>8.00	>8.00	>8.00	0.20	0.50
*Aspergillus versicolor*	8.00	8.00	>8.00	>8.00	0.20	0.50
*Aspergillus niger*	4.00	8.00	>8.00	>8.00	0.20	0.50
*Penicillium funiculosum*	8.00	8.00	>8.00	>8.00	0.20	0.50
*Penicillium ochrochloron*	1.00	1.00	>8.00	>8.00	0.20	0.50
*Trichoderma viride*	4.00	8.00	>8.00	>8.00	0.20	0.30

MIC: Minimal inhibitory concentration; MBC: Minimal bactericidal concentration; MFC: Minimal fungicidal concentration. For antioxidant activity, statistical differences (*p* < 0.05) were found between optimal and normal extracts.

## Supplementary Materials

Table S1: Anthocyanin identification and content in passion fruit epicarp. The retention time (Rt), wavelength of maximum absorption in the visible region (λ_max_), and mass spectral data are presented, Figure S1: Passion fruit epicarp anthocyanin profile extracted at 520 nm. The identified peak corresponds to cyanidin-3-*O*-glucoside, Figure S2: Diagram of the different steps carried out to optimize the extraction conditions for recovery of anthocyanins from passion fruit epicarp.
