# Supplementary material for: Recovery of Anthocyanins from Passion Fruit Epicarp for Food Colorants: Extraction Process Optimization and Evaluation of Bioactive Properties
Source: Molecules. 2020 Jul 14;25(14):3203. doi: 10.3390/molecules25143203 (PMC7397062; doi:10.3390/molecules25143203)
Supplement: Supplementary file 1 [file molecules-25-03203-s001.pdf]

molecules

Supplementary Materials

Recovery of Anthocyanins from Passion Fruit Epicarp for Food Colorants: Extraction Process Optimization and Evaluation of Bioactive Properties

Bejaoui Ghada [1,2], Eliana Pereira [1,*], José Pinela [1], Miguel A. Prieto [3], Carla Pereira [1], Ricardo C. Calhelha [1], Dejan Stojkovićd [4], Marina Sokóvić [4], Khalil Zaghdoudi [2], Lillian Barros [1,*] and Isabel C. F. R. Ferreira [1]

[1] Centro de Investigação de Montanha (CIMO), Instituto Politécnico de Bragança, Campus de Santa Apolónia, 5300-253 Bragança, Portugal; bejghada94@gmail.com (B.G.); jpinela@ipb.pt (J.P.); carlap@ipb.pt (C.P.); calhelha@ipb.pt (R.C.C.); iferreira@ipb.pt (I.C.F.R.F.)

[2] Tunisia Private University (ULT), 32 Bis Av. Kheireddine Pacha, 1002 Tunis, Tunisia; khalilo.zg@gmail.com

[3] Nutrition and Bromatology Group, Faculty of Food Science and Technology, University of Vigo, Ourense Campus, E32004 Ourense, Spain; michaelumangelum@gmail.com

[4] Institute for Biological Research "Siniša Stanković"- National Institute of Republic of Serbia, University of Belgrade, Bulevar Despota Stefana 142, 11000 Belgrade, Serbia; dejanbio@ibiss.bg.ac.rs (D.S.); mris@ibiss.bg.ac.rs (M.S.)

* Correspondence: eliana@ipb.pt (E.P.); lillian@ipb.pt (L.B.)

Academic editor: Maurizio Battino
Received: 5 June 2020; Accepted: 12 July 2020; Published: date

Abstract: The potential of passion fruit (*Passiflora edulis* Sims) epicarp to produce anthocyanin-based colorants with bioactive properties was evaluated. First, a five-level three-factor factorial design coupled with response surface methodology was implemented to optimize the extraction of anthocyanins from dark purple epicarps. The extraction yield and cyanidin-3-*O*-glucoside content were used as response criteria. The constructed models were fitted to the experimental data and used to calculate the optimal processing conditions (t = 38 min, T = 20 °C, S = 0% ethanol/water (*v*/*v*) acidified with citric acid to pH 3, and $R_{S/L}$ = 50 g/L) that lead to maximum responses (3.4 mg/g dried epicarp and 9 mg/g extract). Then, the antioxidant, antimicrobial, and cytotoxic activities of anthocyanin extracts obtained using the optimized method and a conventional extraction method were evaluated in vitro. The extract obtained by the optimized method revealed a higher bioactivity, in agreement with the higher cyanidin-3-*O*-glucoside content. This study highlighted the coloring and bioactive potential of a bio-based ingredient recycled from a bio-waste, which promotes a sustainable bioeconomy in the agri-food sector.

Keywords: *Passiflora edulis* Sims; anthocyanins; extraction optimization; natural colorants; antioxidant activity; oxidative hemolysis; antimicrobial activity

Table S1. Anthocyanin identification and content in passion fruit epicarp. It is presented the retention time (Rt), wavelength of maximum absorption in the visible region (λ_{max}), and mass spectral data.

Peak	Rt (min)	λ_{max} (nm)	$[M+H]^+$ (*m*/*z*)	MS^2 (*m*/*z*)	Tentative identification	Content (mg/g E)
1	20.03	520	449	287(100)	Cyanidin-3-*O*-glucoside	8.3 ± 0.1

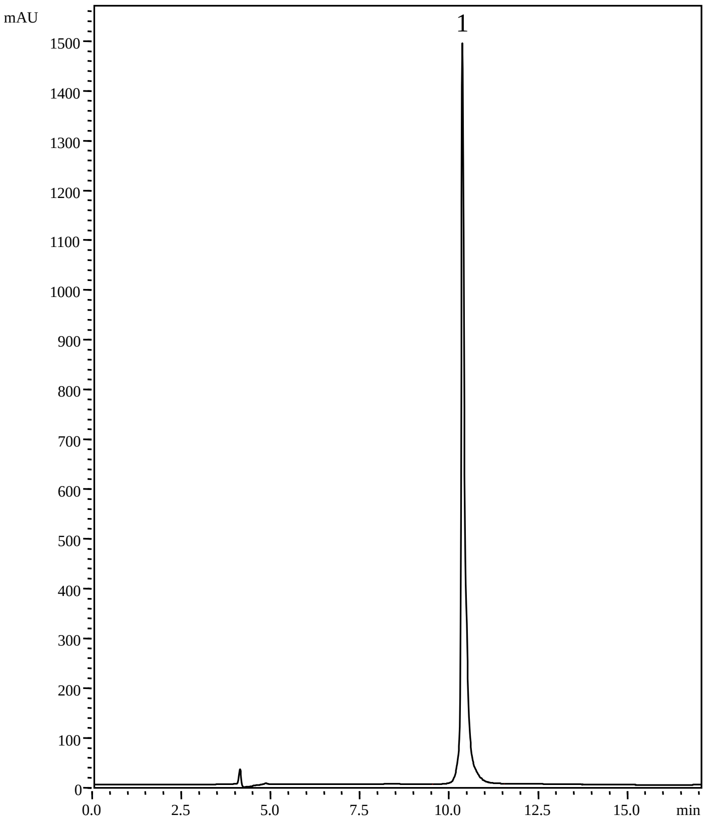

Figure S1. Passion fruit epicarp anthocyanin profile extracted at 520 nm. The identified peak corresponds to cyanidin-3-*O*-glucoside.

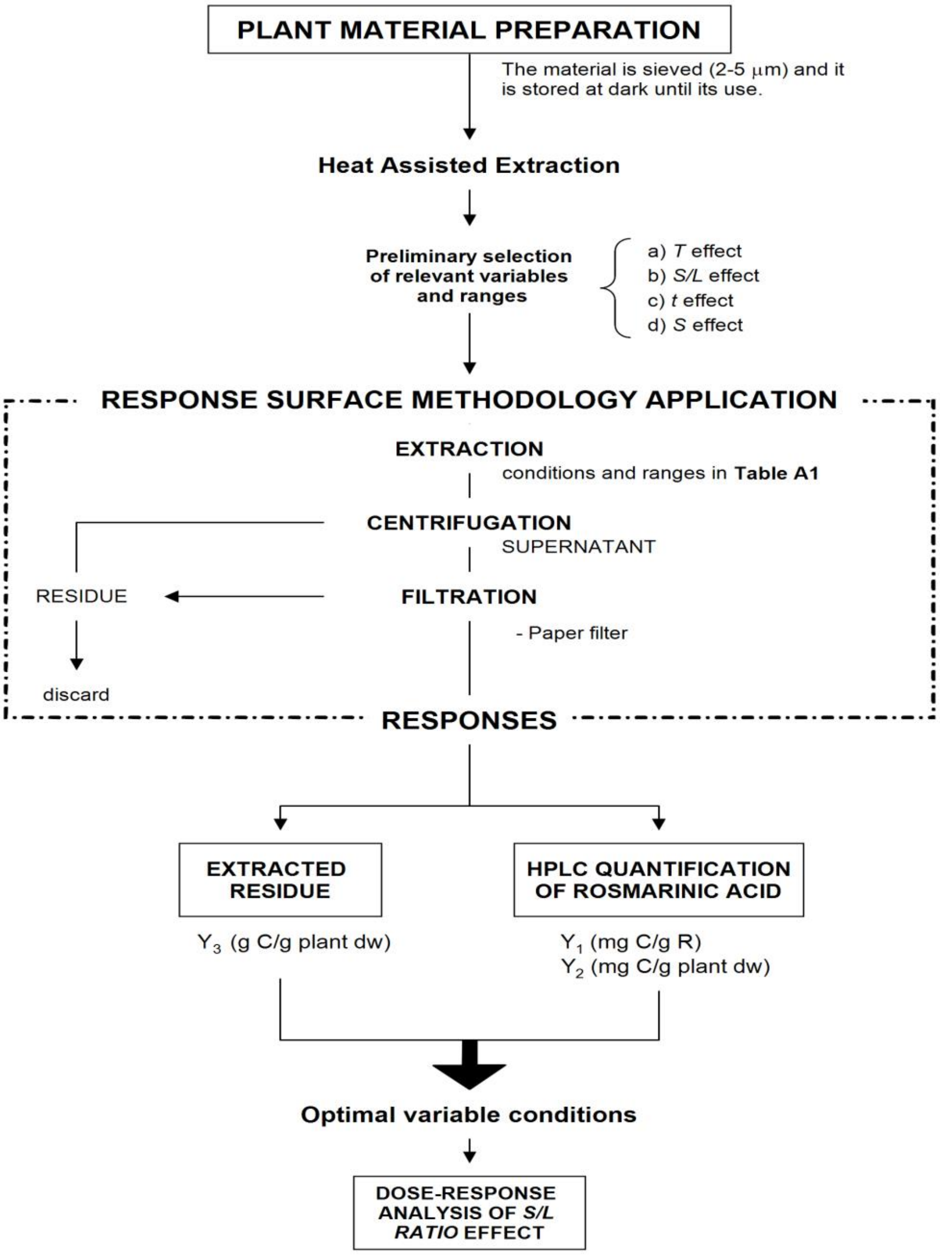

Figure S2. Diagram of the different steps carried out to optimize the extraction conditions for recovery of anthocyanins from passion fruit epicarp.

© 2020 by the authors. Submitted for possible open access publication under the terms and conditions of the Creative Commons Attribution (CC BY) license (http://creativecommons.org/licenses/by/4.0/).
